# Reform in Welfare Teaching

**Published:** 1919-07-19

**Authors:** 


### REFORM IN WELFARE TEACHING.

WE have received a copy of the report, issued after the recent meeting at the Local Government Board under the chairmanship of Sir George Newman, of a conference at which advantage was taken of the presence in this country of several physicians belonging to the staff of pediatric divisions of medical schools in the Dominions and the U.S.A. There is much of interest in this small publication, not perhaps from any great novelty in the opinions expressed, but from the fact that one appendix concisely defines the details of actually working schemes of maternity and child welfare work in England and Wales, forming thus a most useful reference at a time when these matters are under such general discussion. Also the authorities of our medical schools are urged to improve the courses of training which have for so long a time sufficed.

MORE REFINED REQUIREMENTS.

We have on several occasions published articles in the journal, coming actually from quite separate authorities, in an endeavour to point out how sadly deficient has been the practical training in the more refined requirements of obstetrics and gynæcology in spite of the widespread opportunities which inevitably occur in both hospital and private practice. To this we might well add the lack of reasoned dissemination of the elementary principles of dealing with child welfare problems. We would urge consideration of the normal infant and the causes which lay the foundations of disease, rather than by approaching the subject from the old-time pathological standpoint.

At this conference the views expressed very definitely emphasised the importance of breast-feeding, the impropriety of suckling an infant too often, and that practical as well as theoretical instruction in infant hygiene should form an essential part of the training of all persons connected with public welfare work. The policy of limiting maternity work to fully-trained workers, preferably certified midwives, was also strongly advised, but probably one of the most interesting lessons is to be derived from the account given of the medical curriculum followed at Toronto University.

A CANADIAN EXAMPLE.

There the fifth year of medical study is devoted to clinical work entirely, and at least three months are given to pediatrics alone, during which time the infants are not only seen at the children's hospital, but must be followed by the student through their home life as well, visits being made to the local houses from day to day. Such is a poor outline of the thoroughness with which the problem is being tackled overseas, and the two resolutions adopted at the close of the conference indicate more clearly perhaps the trend of these developments. In the first place, "Every medical student preparing for a registrable qualification shall receive adequate training in the subjects of infancy and childhood in health and disease; that attendance in a department where instruction is given in these subjects should be compulsory for a period of not less than three months; and that some special part of the final examination in medicine should be devoted to these subjects." In the second, "this study should follow upon and be co-ordinated with a satisfactory course in obstetrics and gynæcology, and should be made available for post-graduate students."

